# Insights on the in-vitro binding interaction between donepezil and bovine serum albumin

**DOI:** 10.1186/s13065-023-00944-z

**Published:** 2023-04-06

**Authors:** Reem N. El Gammal, Heba Elmansi, Ali A. El-Emam, Fathalla Belal, Perihan A. Elzahhar, Ahmed S. F. Belal, Mohammed E. A. Hammouda

**Affiliations:** 1 Department of Medicinal Chemistry, Faculty of Pharmacy, Mansoura University, Mansoura, 35516 Egypt; 2 Pharmaceutical Analytical Chemistry Department, Faculty of Pharmacy, Mansoura University, Mansoura, 35516 Egypt; 3 Department of Pharmaceutical Chemistry, Faculty of Pharmacy, Alexandria University, Alexandria, 21521 Egypt; 4 Department of Pharmaceutical Chemistry, Faculty of Pharmacy, Horus University - Egypt (HUE), New Damietta, Egypt

**Keywords:** Donepezil, Bovine serum albumin, Synchronous fluorimetry, Fourier transform infrared, Fluorescence resonance energy transfer, Molecular docking

## Abstract

In this work, the binding mechanism between donepezil (DNP) and bovine serum albumin (BSA) was established using several techniques, including fluorimetry, UV- spectrophotometry, synchronous fluorimetry (SF), fourier transform infrared (FTIR), fluorescence resonance energy transfer (FRET) besides molecular docking study. The fluorescence quenching mechanism of DNP-BSA binding was a combined dynamic and static quenching. The thermodynamic parameters, binding forces, binding constant, and the number of binding sites were determined using a different range of temperature settings. Van't Hoff's equation was used to calculate the reaction parameters, including enthalpy change (ΔH^ο^) and entropy change (ΔS^ο^). The results pointed out that the DNP-BSA binding was endothermic. It was shown that the stability of the drug-protein system was predominantly due to the intermolecular hydrophobic forces. Additionally, the site probing method revealed that subdomain IIA (Site I) is where DNP and BSA's binding occurs. This was validated using a molecular docking study with the most stable DNP configuration. This study might help to understand DNP's pharmacokinetics profile and toxicity as well as provides crucial information for its safe use and avoiding its toxicity.

## Introduction

The major plasma protein, serum albumin, facilitates the movement of both exogenous and endogenous small molecules throughout the circulatory system by binding at particular sites and forming molecular interactions with them [1]. It was reported that there are similarities in the structure of BSA and human serum albumin (HSA), making it a crucial topic for in-vitro study. BSA has a molecular weight of 66.5 kDa and is made up of 583 amino acids. BSA's protein structure is split into three subdomains organized in a linear configuration and further segmented into A and B [2–4]. Sites I, II, or III, found in the hydrophobic cavities of subdomains IIA and IIIA, respectively, are available for endogenous and exogenous ligands to bind to BSA. The produced stable complex is crucial for gaining fundamental identifications of the binding between plasma and drugs [5].

The type of a drug's interaction with plasma proteins and serum albumins largely determines its pharmacokinetics [6, 7]. Both the drug's concentrations in its free and complex forms are essentially controlled by its affinity for serum albumin, whereas their equilibrium is essential for the drug's mode of action. Subsequently, it is critical to fully understand the drug's binding method with serum albumin [8].

Alzheimer's disease (AD) is a progressive neurodegenerative disorder characterized by many pathological features, the most important of which is cholinergic deficit [9]. The main target of the available therapeutic options for AD is the improvement of CNS cholinergic functions. DNP is one of those drugs. It is an acetylcholinesterase inhibitor (AChEI) that leads to the accumulation of Acetylcholine which is deficient in AD and dementia patients. DNP has previous reports of its significant efficacy in treating memory loss and cognitive impairment in AD patients [10]. DNP is recognized by the American Geriatrics Society's Beer's list as a high-risk medication in elderly patients because of the population's higher incidence of orthostatic hypotension and bradycardia [11]. Higher doses of DNP can cause toxicity that manifests in colitis and beta-blocker overdose likewise. Symptoms are nausea, vomiting, anorexia, diarrhea, fatigue, and dizziness. Other common adverse effects include abdominal pain, dyspepsia, rash, pruritus, headache, muscle cramps, insomnia, sweating, tremor, and syncope; upper respiratory tract and urinary tract infections have been noted [12]. The gastrointestinal tract absorbs DNP effectively. It is about 95% bound to plasma proteins, mainly albumin [12]. Additionally, because the liver is where the drug is processed, hepatic impairment worsens with aging owing to decreased liver volume and hepatic blood flow [12]. So DNP and other acetylcholinesterase inhibitors should be used with caution in patients who suffer from chronic diseases [12]. To avoid the drug's harmful effects and toxicity, it appears critical to investigate its binding process with serum albumin.

**Fig. 1:**
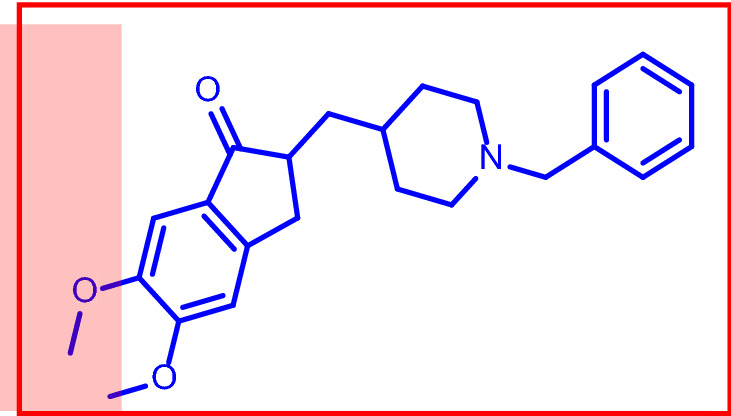
Chemical structure of donepezil (DNP)

Based on this literature, the interaction between DNP (Fig. 1) and BSA was investigated in this in-vitro study utilizing the quenching fluorescence technique. Using Tris buffer (pH 7.4) at the simulated physiological conditions, thermodynamic characteristics were utilized to derive binding constants at three different temperatures. Furthermore, the binding forces and binding sites were detected. Ultraviolet absorbance, SF, and FTIR were also utilized to analyze BSA structural alterations caused by DNP binding, and the results were confirmed by molecular docking.

## Experimental

### Chemicals and reagents

DNP was generously supplied by Delta Pharmaceutical Industries, 10th of Ramadan City, Egypt. Diazepam was provided by Amoun Pharmaceutical Co., Cairo, Egypt. Indomethacin was provided by Medical Union Pharmaceuticals Co, Ismailia, Egypt.

BSA was purchased from Sigma Aldrich Co. (ChemieGmbh, Munich, Germany) with batch No. SLBM6044V and molecular weight of 66.5 kDa. Tris (hydroxymethyl) aminomethane hydrochloride (Tris–HCl) was purchased from Sigma Aldrich (Germany).

### Instruments and software

#### Fluorescence measurements

Cary Eclipse Fluorescence Spectrophotometer with Xenon flash lamp from Agilent Technologies (USA) was employed using a high detector voltage (900 V) and smoothing factor of 20.

When recording the fluorescence spectra, it is expected that the inner filter effect (IFE) is necessarily measured during fluorescence measurements [13]. The fluorescence intensity (FI) as a consequence of IFE was adjusted using the equation below [14]:

$$ F_{cor} = F_{obs} \times e^{{(A_{ex} + A_{em} )/2}} $$

F_cor_: the corrected FI, F_obs_: observed FI, A^ex^ and A^em^ are the summations of DNP absorbance at excitation 285 nm and the emission wavelength (λ), respectively.

#### For UV–Vis spectroscopic measurements

Spectrophotometric measurements were conducted using Shimadzu (Kyoto, Japan) UV-1601 PC, UV–Visible double-beam spectrophotometer.

#### Fourier-transform infrared spectroscopy (FT-IR)

FT-IR spectra were displayed using Thermo Fisher Scientific Nicolet—iS10 FT-IR Spectrometer equipped with a Ge/KBr beam splitter and a DTGS detector from 4000 to 1000 cm^−1^. In our experiments, 32 scans were recorded at 4 cm^−1^ resolution.

Hanna pH Meter (Romania) was used to adjust the pH values.

#### Molecular docking experiment

The docking study was carried out using Molecular Operating Environment (MOE 2019.0102) software (Chemical Computing Group, Montreal, Canada) using the crystal structure of the active site of bovine serum albumin in complex with 3,5-diiodosalicyclic acid (PDB **4JK4**). Hydrogen atoms were added to DNP, partial charges were estimated, and Force Field MMFF94x was used to aid energy minimization. Unwanted protein chains, water molecules, and surfactant molecules were also omitted. Besides, the protein was prepared using QuickPrep panel, which comprised structure preparation, protonate 3D, receptor tethering, and refinement to RMS gradient of 0.1 kcal/mol/A and keeping all other default parameters. In the Dock panel, receptor and site fields were set to receptor and ligand atoms, respectively. MOE's default settings were implemented including the triangle matcher as a placement mode and London dG as the principal scoring function, along with GBVI/WSA dG scoring as an auxiliary refining step and retaining 30 initial poses followed by 5 final poses after refinement. In addition, the resultant database was examined for different poses using the MOE browser. The best configurations based on both the binding interactions and docking score were chosen. This was accomplished via the use of scoring algorithms and a visual examination of the hydrogen bonding, hydrophobic, and ionic interactions with the active site residues.

### Procedures

#### Stock solutions preparation

Both BSA (2 × 10^–6^ M) and DNP (10^–4^ M) stock solutions were daily prepared in bidistilled water. Tris buffer (20 mM) was prepared by dissolving in bidistilled water, and pH was modified to 7.4 using 1.0 M HCl*.* The prepared solutions were stored in the refrigerator at 4 °C and further diluted to the appropriate working solutions.

#### UV–Vis spectroscopic measurements

During this procedure, BSA concentration was kept unchanged at 4 × 10^–7^ M, whereas the concentration of the drug was altered from 0.4 × 10^–5^ to 5.5 × 10^–5^ M (0.4, 0.5, 1, 1.5, 2, 2.5, 3, 4, 5, 5.5 × 10^–5^ M). Tris buffer was used to complete the volume of solutions and was kept constant at temp 298 K. UV absorption of the prepared solutions was scanned in the region of 190–350 nm. To eliminate interference likely to be encountered from the solvent, the spectra were corrected through subtraction of the absorption of solvent from the absorption of each BSA and BSA-DNP complex [15].

#### Quenching measurements

BSA fluorescence quenching was investigated in Tris buffer at three different temperatures: 298, 310, and 318 K over the wavelength range 280 to 500 nm after excitation at 285 nm. The concentration of BSA was 4 × 10^–8^ M while increasing DNP concentration from 0.4 × 10^–5^ M to 4 × 10^–5^ M (0.4, 0.6, 1.4, 1.6, 2, 2.2, 3, 3.2, 3.6, 4 × 10^–5^ M). The resulting emission spectra were scanned.

#### Synchronous fluorescence measurements

The SF spectra of BSA in Tris buffer of pH 7.4 were scanned in the range of 200–400 nm with different concentrations of DNP (0–4 × 10^–5^ M). The study was conducted using Δλ = 60 nm and Δλ = 15 nm for tryptophan and tyrosine residues, respectively. The resulting SF spectra in both cases were recorded.

#### FTIR method

The FTIR spectra of BSA (5 × 10^–7^ M) with and without DNP were recorded from 1500 to 1800 cm^−1^ and adjusting BSA: drug molar ratio of 1:1. In addition, the absorbance values of Tris and free DNP were recorded and subtracted from BSA-DNP complex spectra.

#### Site markers competitive binding

The competitive binding methods were established by employing two well-known site markers: indomethacin (site I) and diazepam (site II). The procedure involved combining BSA and one of the site markers in equal amounts, then letting them rest for 30 min to confirm complete interaction. Subsequently, the combination was treated with various DNP concentrations, and the emission fluorescence spectra were recorded.

## Results and discussion

### UV–visible spectrophotometry

**Fig. 2:**
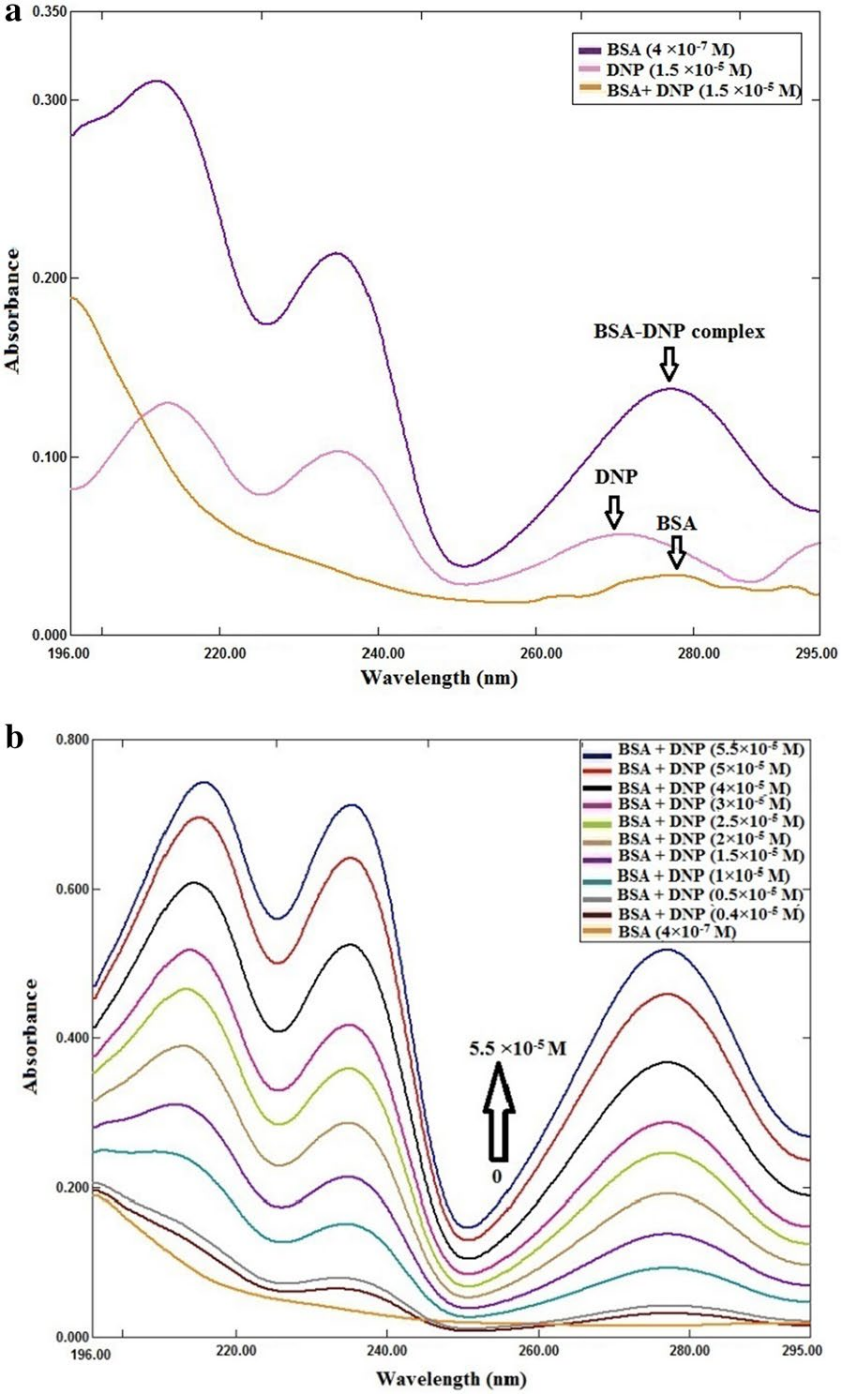
**a** Ultraviolet absorption spectra of BSA (4 × 10^–7^ M), DNP (1.5 × 10^–5^ M) and BSA-DNP complex (1.5 × 10^–5^ M) at *T* = 298 K, *pH* = 7.4. **b** Ultraviolet absorption spectra of BSA (4 × 10^–7^ M) with different concentrations of DNP (× 10^–5^ M): (0.4, 0.5, 1, 1.5, 2, 2.5, 3, 4, 5, 5.5) at *T* = 298 K, *pH* = 7.4

Absorption in the UV–visible region was utilized as a reliable method to study protein structural alterations and its complex formation with drugs [16–18]. UV absorption of the prepared solutions was scanned in the region of 190–350 nm. To eliminate interference likely to be encountered from the solvent, the spectra were corrected through subtraction of the absorption of solvent from the absorption of each BSA and BSA-DNP complex. DNP has a significant absorption peak at 270 nm. The spectrum of protein is affected by the microenvironment around the chromophores [19]. There is a significant absorption peak at 278 nm due to the aromatic amino acids (tryptophan, tyrosine, and phenylalanine), as illustrated in Fig. 2a. The increment in peak intensity with increasing concentrations of DNP illustrated in Fig. 2b signifies a possible binding between DNP and BSA. This in turn causes the loosing and unfolding of the protein backbone with decreasing in the hydrophobicity of its microenvironment.

### Spectrofluorimetric method

**Fig. 3:**
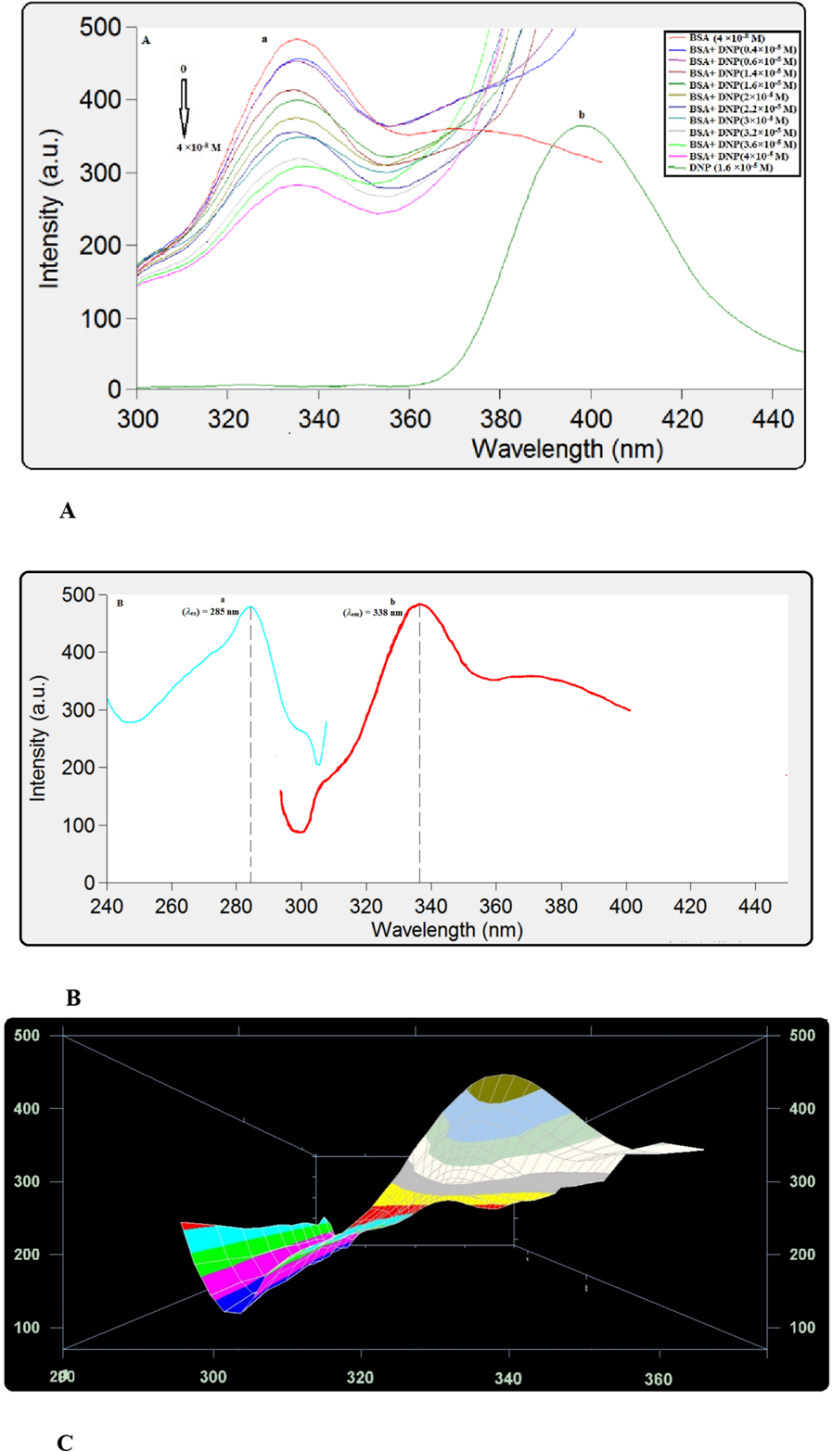
**A** **a** Effect of DNP concentrations (× 10^–5^ M): (0.4, 0.6, 1.4, 1.6, 2, 2.2, 3, 3.2, 3.6, 4) on the fluorescence spectra of BSA (4 × 10^–8^ M) while **b** emission spectra of DNP (1.6 × 10^–5^ M) at *T* = 298 K, *pH* = 7.4. **B** Excitation (a) and emission (b) spectra for BSA (4 × 10^–8^ M) at *T* = 298 K and *pH* = 7.4, and (λ_ex_) = 285 nm. **C** BSA (4 × 10^–8^ M) 3D Fluorescence spectra in presence of DNP (× 10^–5^ M): (0.4, 0.6, 1.4, 1.6, 2, 2.2, 3, 3.2, 4) at *T* = 298 K, *pH* = 7.4

This method is developed to study the drug–protein binding processes and learn more about the binding constant, the number of binding sites, and thermodynamic parameters. Mostly, alterations in the FI of the protein are monitored at the maximum emission wavelength [13, 20]. Figure 3A, B and C demonstrate the quenching of BSA fluorescence emission when DNP concentrations are increased. BSA has 18 tyrosine residues and two tryptophan residues. They display a strong band when excited at 285 nm [21].

When DNP is added to the BSA solution, a DNP-BSA complex is formed, which causes the latter's fluorescence to be quenched. The Stern–Volmer equation was utilized to evaluate the binding parameters at various temperatures adopting the following equation [13]:

1 $$\frac{{F}_{0}}{F}=1+ {K}_{sv}\left[Q\right]$$

F_ο_ and F are FI of BSA in the absence and presence of DNP, K_SV_ is the Stern–Volmer quenching constant, [Q] is the molar concentration of DNP.

**Fig. 4:**
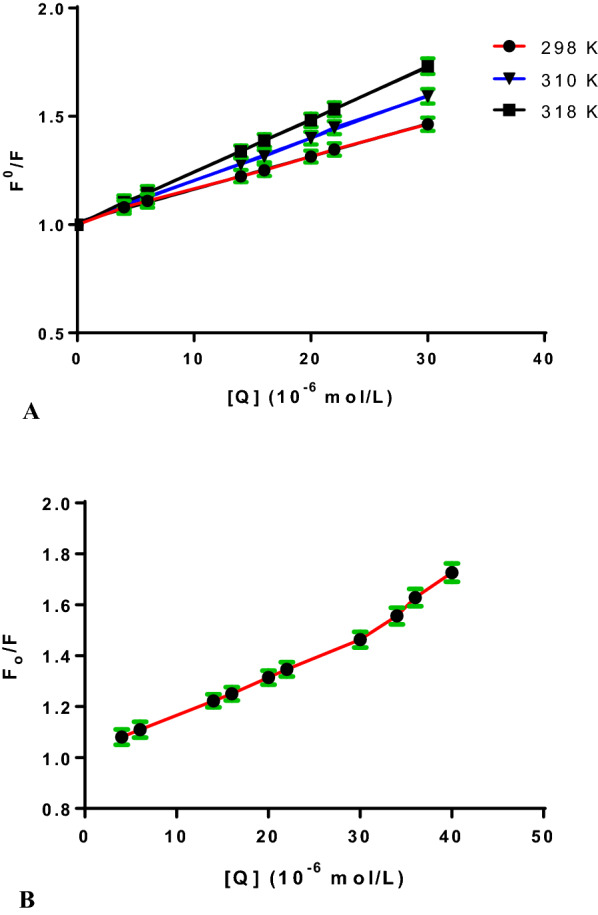
**A** The linear Stern–Volmer plots for the BSA-DNP system at different temperatures. **B** Stern–Volmer plots of the fluorescence quenching of the BSA-DNP system

The Stern–Volmer constant could be calculated using the plot of Eq. 1 (shown in Fig. 4A). Fluorescence can be quenched via one of three mechanisms: static, dynamic, or a mixture of the two. As both systems are temperature-dependent, their principles differ significantly [13, 22]. In the dynamic quenching process, quenching constants are expected to rise upon increasing the temperatures leading to high diffusion coefficients. Meanwhile, greater temperatures might reduce complex stability, resulting in a reduction in the static quenching constant values [23, 24]. The formation of this complex is also demonstrated by the values of the quenching rate constant (k_q_). Generally, the dynamic quenching of fluorescence is described by the Stern–Volmer equation [13]

2 $$\frac{{F}_{0}}{F}=1+{K}_{sv}[Q]=1+ {k}_{q}{\tau }_{0}\left[Q\right] $$

where F_ο_ and F denote the steady-state FI of BSA in the absence and presence of DNP, K_SV_ is the Stern–Volmer quenching constant, [Q] is the molar concentration of DNP, *k*_*q*_ is the bimolecular quenching rate constant, *τ*_*ο*_ is the average lifetime of the fluorophore in the excited state for a biomolecule (10^–8^ s) [13, 25, 26].

Obviously, the dependence of F_o_/F on [Q] is linear for dynamic and static quenching. However, for the combined dynamic and static quenching, the characteristic feature of the dependence of F_o_/F on [Q] is an upward curvature, concave towards the Y-axis. And the dependence of F_o_/F on [Q] is described by the following modified Stern–Volmer equation [13]:

3 $$\frac{{F}_{o}}{F}=\left(1+{K}_{D}\left[Q\right]\right)\left(1+{K}_{S}\left[Q\right]\right)=1+\left({K}_{D}+{K}_{S}\right)\left[Q\right]+{K}_{D}{K}_{S}[{Q]}^{2}$$

where K_S_ and K_D_ are the static and dynamic quenching constants, respectively.

From Fig. 4B, the dependence of F_o_/F on [Q] is nonlinear in the concentration range of DNP from (0.4 × 10^–5^ M to 4 × 10^−5^ M), indicating that the quenching mechanism of BSA induced by DNP may be the combined dynamic and static quenching. Meanwhile, it can be observed that the dependence of F_o_/F on [Q] is closely linear when the concentrations of DNP are lower than 3 × 10^–5^ M, which is treated according to the Stern–Volmer equation and the results are listed in Table 1, it can be found that the K_SV_ values increased with the increasing temperature, suggesting that the quenching mechanism is initiated by dynamic quenching. While the bimolecular quenching rate constant of the complex was found to be (1.47 × 10^12^ L mol^−1^ s^−1^), which is larger than the maximum scatter collision quenching constant, k_q_ of different quenchers with the biopolymer (2 × 10^10^ L mol^−1^ s^−1^) pointing out to a static quenching during the interaction between DNP and BSA [24, 27–30].

**Table 1:** The studied DNP-BSA interaction parameters at different temperatures

T (K)	K_sv_ × 10^4^ (L mol^−1^)	k_q_ × 10^12^ (L mol^−1^ s^−1^)	r^2^
298	1.47	1.47	0.9999
310	1.86	1.86	0.9994
318	2.24	2.24	0.9994

### Estimation of the binding constant and binding site

**Fig. 5:**
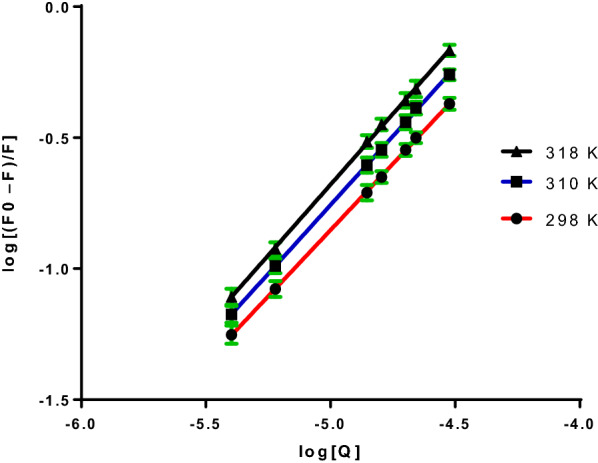
Plots of log (F_o_–F)/F versus Log [Q] at three different temperatures

The affinity of the drug-receptor is one of the important indications for evaluating the effects of drug, which relates to the binding constant (*K*_b_). Both binding constant and binding sites are calculated using the so-called Modified Stern–Volmer equation [31]:

4 $$\mathrm{log}\frac{{F}_{o}-F}{F}=\mathrm{log}{K}_{b}+n log \left[Q\right]$$

where K_b_ and n are the binding constant and number of the binding site, respectively. By graphing the relationship between log ($\frac{{F}_{o}-F}{F}$) and log [Q] as illustrated in Fig. 5, a linear relationship determined the values of n and log *K*_*b*_ from the slope and intercept, respectively. Table 2 displays the findings achieved at different temperatures. It was found that BSA and DNP bind approximately in a ratio of 1:1 and that the binding constant increased with increasing temperature.

**Table 2:** DNP-BSA complex binding characteristics at various temperatures

T (K)	k_b_ × 10^4^ (Lmol^−1^)	n	r^2^
298	1.472	1.000	0.9999
310	3.027	1.044	0.9998
318	5.248	1.076	0.9998

### Thermodynamicscharacteristics and binding forces

The resulting complexes can be formed through four leading forces: electrostatic interactions, hydrogen bonding, hydrophobic, and Van der Waals forces [28]. The reaction's binding forces are defined by the values of the thermodynamic parameters: enthalpy change (ΔH^ο^) and entropy change (ΔS^ο^). Ross and Subramanian explored the relationship between alterations in thermodynamic parameters and binding forces [32]. The positive values of ΔH^ο^ and ΔS^ο^ demonstrated that hydrophobic forces are thepredominant, whereas Van der Waals forces and hydrogen bonding predominate when ΔH^ο^ < 0 and ΔS^ο^ < 0. When ΔH^ο^ < 0 and ΔS^ο^ > 0, electrostatic forces are regarded as the main forces. In cases of small temperature changes, Van't Hoff equation can be used for the determination of both ΔH^ο^ and ΔS^ο^ [32, 33].

5 $$\mathrm{ln}{K}_{b}=-\frac{{ \Delta \mathrm{H}}^{\mathrm{o}}}{\mathrm{RT}}+\frac{{\Delta \mathrm{S}}^{\mathrm{o}}}{\mathrm{R}}$$

*K*_b_ is the binding constant at temperature T and R is the gas constant.

**Fig.6:**
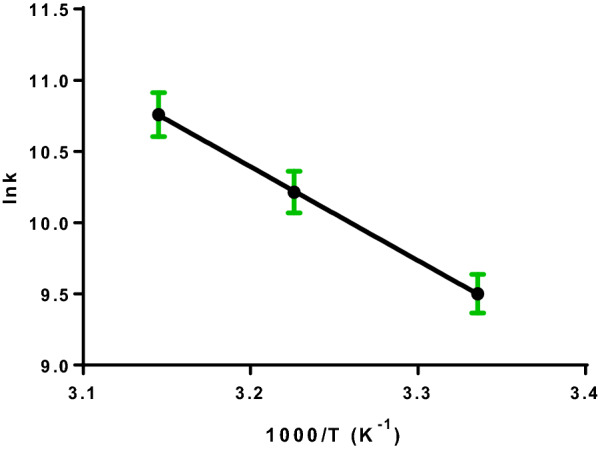
Van't Hoff plot for BSA-DNP binding

By plotting ln*K*_*b*_ versus 1/*T* as displayed in Fig. 6, a linear relationship was established with slope representing the enthalpy change (ΔH^ο^), and intercept representing the entropy change (ΔS^ο^). Consequently, free energy change (ΔG^ο^) can be computed using the below equation:

6 $${\Delta G}^{\mathrm{o}}= {\Delta H}^{\mathrm{o}}-T{\Delta S}^{\mathrm{o}}$$

The negative value of (ΔG^ο^) and the positive (ΔS^ο^) represented in Table 3, demonstrate the spontaneous nature of DNP-BSA binding. Moreover, the key driving force in this interaction is the hydrophobic force. An endothermic reaction is established by relating the positive value of enthalpy change (ΔH^ο^) and the elevated K values upon increasing temperature.

**Table 3:** Thermodynamic parameters of DNP-BSA interaction at pH 7.4

T (K)	Δ*H*^ο^ (kJ mol^−1^)	Δ*G*^ο^(kJ mol^−1^)	Δ*S*^ο^ (J mol^−1^ K^−1^)	r^2^
298	46.22	−24.46	237.2	0.9969
310		−27.31		
318		−29.21		

### Synchronous fluorescence spectroscopy

**Fig.7:**
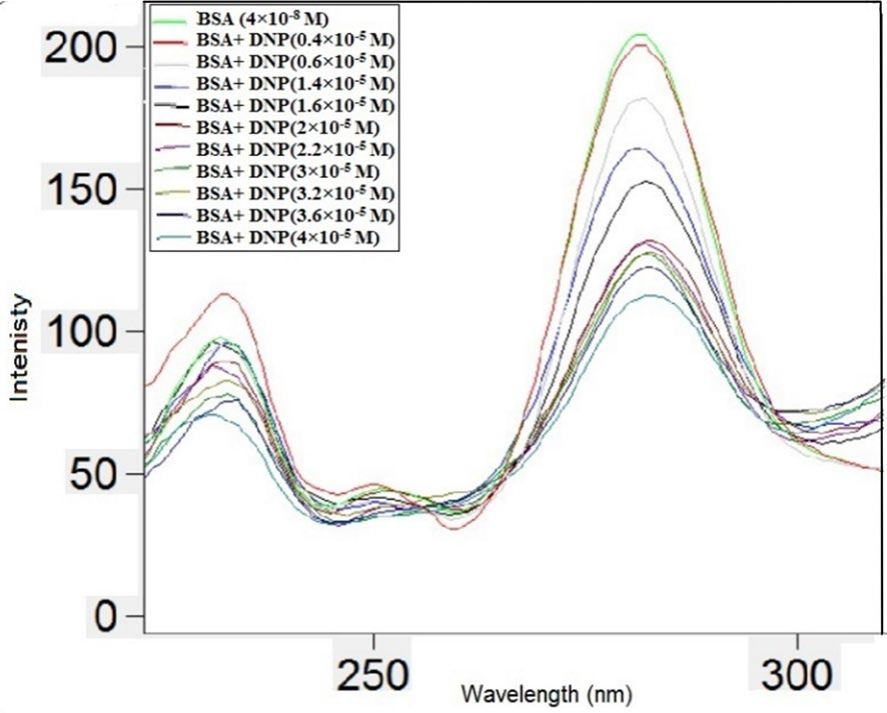
Synchronous fluorescence spectra of BSA (4 × 10^–8^ M) with different concentrations of DNP (× 10^–5^ M): (0.4, 0.6, 1.4, 1.6, 2, 2.2, 3, 3.2, 3.6, 4) at Δλ of 15 nm

**Fig.8:**
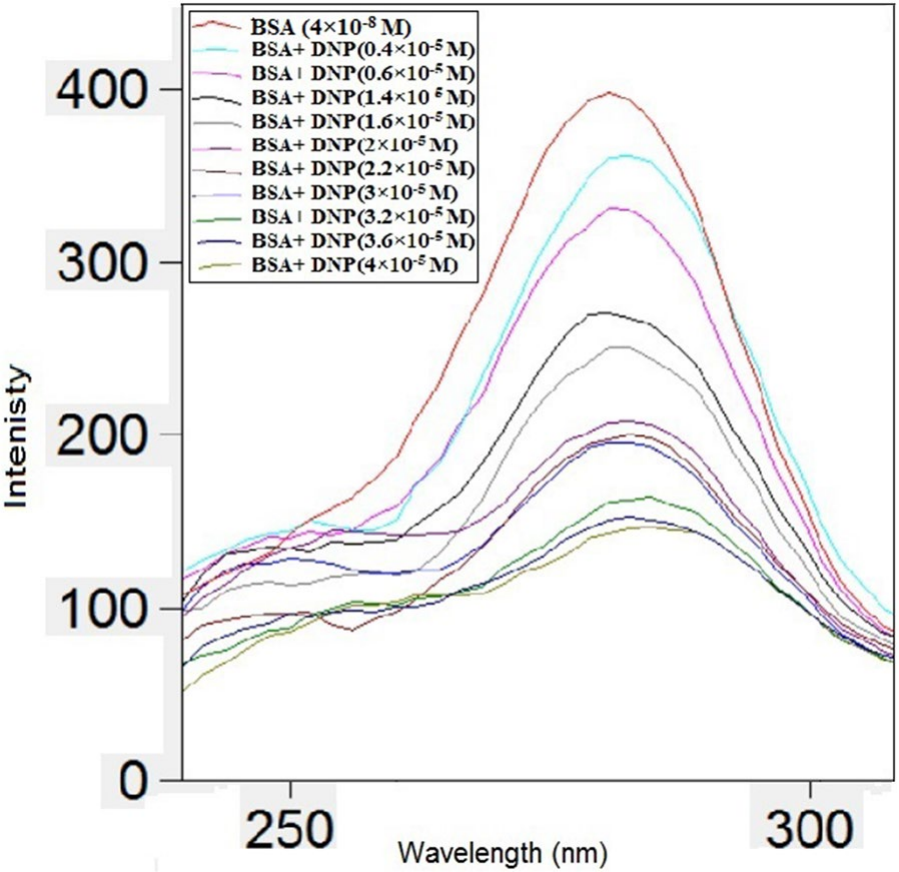
Synchronous fluorescence spectra of BSA (4 × 10^–8^ M) with different concentrations of DNP (× 10^–5^ M): (0.4, 0.6, 1.4, 1.6, 2, 2.2, 3, 3.2, 3.6, 4) at Δλ of 60 nm

Synchronus fluorescence is very valuable for achieving a shift in emission wavelength required to examine the protein's microenvironment [34]. Tryptophan and tyrosin residues in BSA may be identified using SF spectra at (Δλ) = 60 nm and 15 nm, respectively. The SF spectra of Tyr (Fig. 7) and Trp residues (Fig. 8) in BSA with different DNP concentrations are shown. Figure 7 demonstrates that the utilized concentrations kept the maximum emission range at Δλ of 15 nm unaffected, whereas at Δλ 60 nm, a small red shift was detected as displayed in Fig. 8. Tryptophan residues' surrounding polarity was diminished, indicating that the interaction occurred in a hydrophobic environment. The observed spectrum alterations lead to the establishment of BSA's conformation after binding with DNP [35].

### Site markers competitive binding

**Fig. 9:**
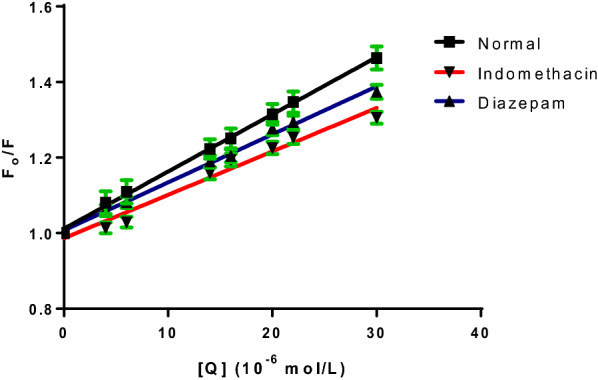
The linear Stern–Volmer plots describing BSA quenching by DNP alone and in presence of site markers (indomethacin and diazepam)

BSA is divided into three domains: I, II, and III, each of which is further separated into two subdomains: A and B [3, 36]. To discover the DNP binding site in BSA, the site marker measures were examined. Sites I and II, which are located in the hydrophobic regions of subdomains IIA and IIIA, respectively, are where drugs bind to albumin, according to Sudlow et al. [37]. The binding sites for indomethacin and diazepam are site I and site II, respectively [38, 39]. Equation 1 is used to calculate the binding characteristics arising from the influence of both site markers on DNP-BSA binding (Fig. 9). Table 4 displays the binding constants of DNP-BSA interaction in the presence of the two site markers. Noticeably, DNP binding with BSA is attenuated by indomethacin. However, the binding constant was not altered by diazepam. So, it may be concluded that site I of subdomain IIA is the location where DNP-BSA binding takes place. These findings are reliable with the outcomes from the molecular docking approach explained in “Molecular docking” section

**Table 4:** Estimated Stern–Volmer constants and bimolecular quenching rate constants for DNP-BSA interaction with and without the addition of site markers

Site marker	K_sv_ (10^4^ L mol^−1^)	Kq (10^12^ L mol^−1^ s^−1^)
BSA + DNP	1.47	1.47
BSA + DNP + IND	0.82	0.82
BSA + DNP + DIA	1.24	1.24

### FTIR spectroscopy

**Fig.10:**
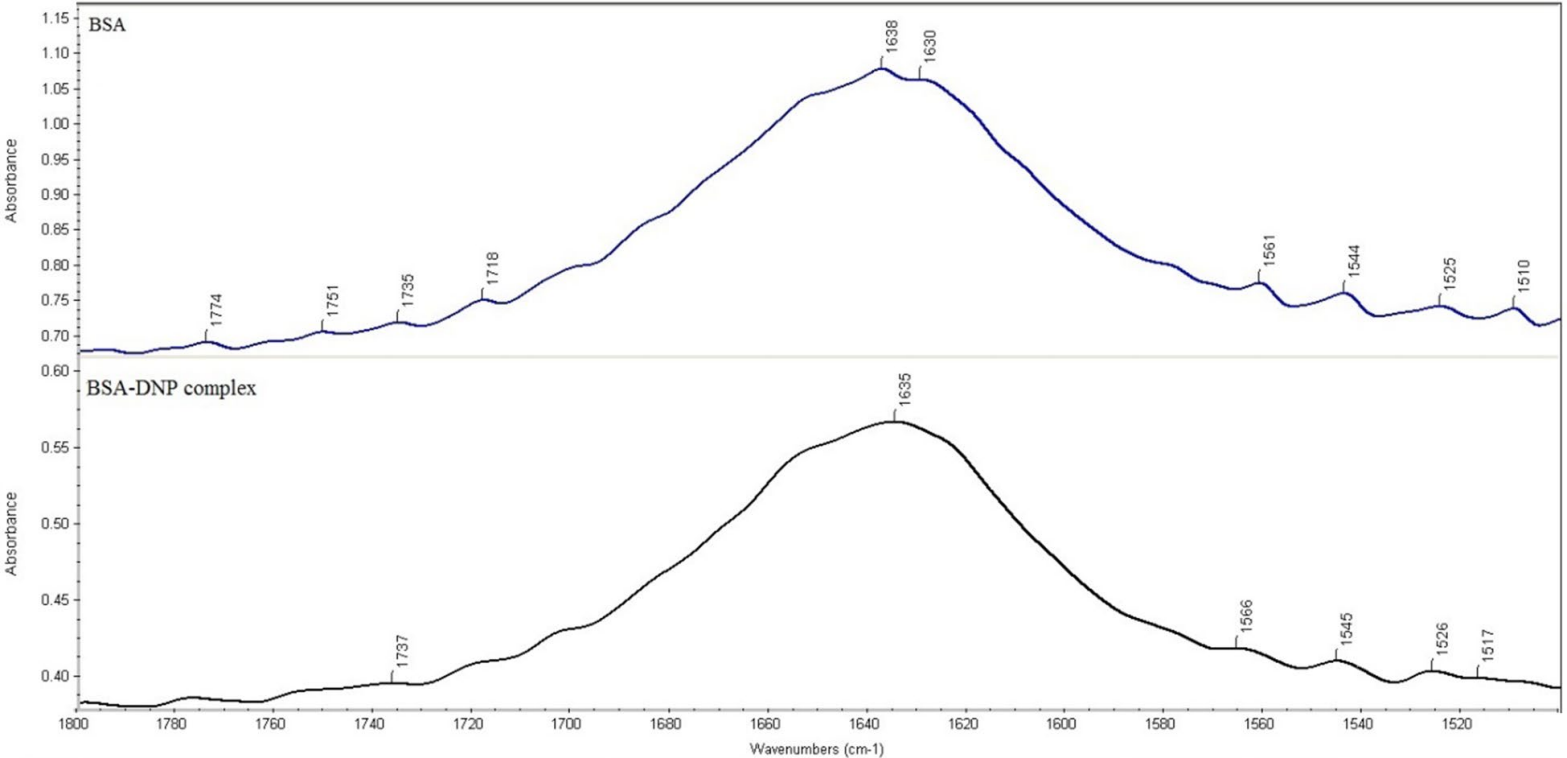
FTIR Spectra of BSA and BSA-DNP complex at 298 K and *pH* of 7.4

Protein dynamics and secondary structures can be studied using FTIR Spectroscopy [40]. The FTIR spectra displayed in Fig. 10 provide evidence of the interaction between DNP and BSA. The amide bands are the constituents of the protein's IR spectrum, as they induce several peptide moieties vibrations. Secondary protein structure studies frequently use the peaks of amides I and II, which are found in the area of 1600–1700 cm^−1^ and 1500–1600 cm^−1^, respectively. It is possible to examine the secondary structures of protein using the band from amide I since it is more vulnerable to changes than the band from amide II. Although there is a slight shift, it indicates that the secondary protein structure is changed during the DNP-BSA interaction since the peak of the amide I position was shifted from 1638 cm^−1^ to 1635 cm^−1^ during the DNP-BSA interaction [41].

### Fluorescence resonance energy transfer (FRET)

**Fig.11:**
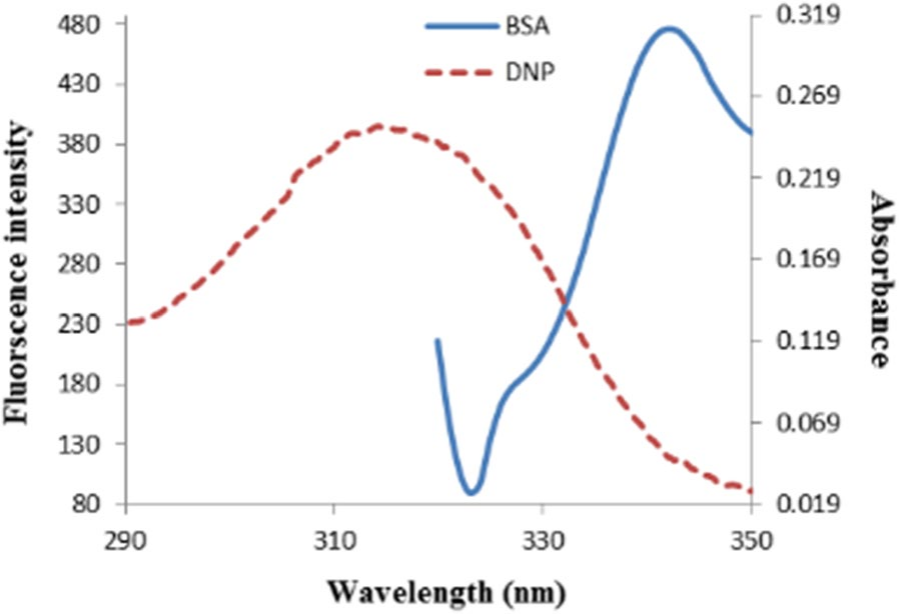
An overlay showing the spectral overlap of the fluorescence emission spectrum of BSA (4 × 10^–8^ M) with the UV absorption spectrum of DNP (2.5 × 10^–5^ M)

In the FRET mechanism, the donor molecule (BSA) and the acceptor molecule (DNP) transmit non-radiative energy over a distance. Resonance happens in a distance-dependent manner without molecule collision or thermal energy conversion through dipole–dipole combining between the two fluorophores. As stated by Fӧrster’s non-radiative energy transfer theories, numerous factors influence FRET [42]:

1. The donor's emission and acceptor's absorption spectra overlap as clarified in Fig. 11.
2. The spectral overlap integral (*J*) signifies the magnitude of this overlap.
3. The transition dipole orientations of the acceptor and donor molecules should be parallel.

The following formula was used to calculate the efficiency of energy transfer (*E*):

7 $$E=1-\frac{F}{{F}_{o}}=\frac{{R}_{o}^{6}}{{R}_{0}^{6}{+ r}^{6}}$$

where *F*_*o*_ and *F*: FI in the absence and presence of DNP, respectively. *R*: the distance between BSA and DNP. *R*_*o*_: the Fӧrster distance where the energy transfer is 50% efficient.

Also, Eq. 8 was used to get the value of R:

8 $$ R_{o}^{6} = 8.8 \times 10^{ - 25} k^{2} N^{ - 4} $$

where *k*^*2*^ is the dipole angular orientation of each molecule, *N* is the refractive index, *Ф* is the fluorescence quantum yield of the donor in absence of the acceptor, *J* is the overlap integral between the fluorescence emission spectrum of the donor and the absorbance spectrum of the acceptor and computed using Eq. 9:

9 $$J=\frac{\int F(\lambda )\varepsilon (\lambda ){\lambda }^{4}\Delta \lambda }{\int F(\lambda )\Delta \lambda }$$

where *F* (λ) is the FI of the fluorescent donor at this wavelength λ. (λ) is the molar absorption coefficient of the acceptor at wavelength λ.

In the current work, *k*^*2*^ = 2/3. *N* = 1.336 and *Ф* = 0.15. The spectral overlap integral (*J*) is computed over the range of 300–450 nm. Values of $J = 5072 \times 10^{ - 14} {\text{cm}}^{{{3}}} \,{\text{L}}\,{\text{mol}}^{{ - 1}}$ [43], *E* = 0.24, *R*_*o*_ = 3.41 nm, and *r* = 4.13 nm can be calculated from Eqs. 7–9. The average distance between BSA and DNP is less than 8 nm proposing the occurrence of energy transfer [44].

### Molecular docking

**Fig. 12:**
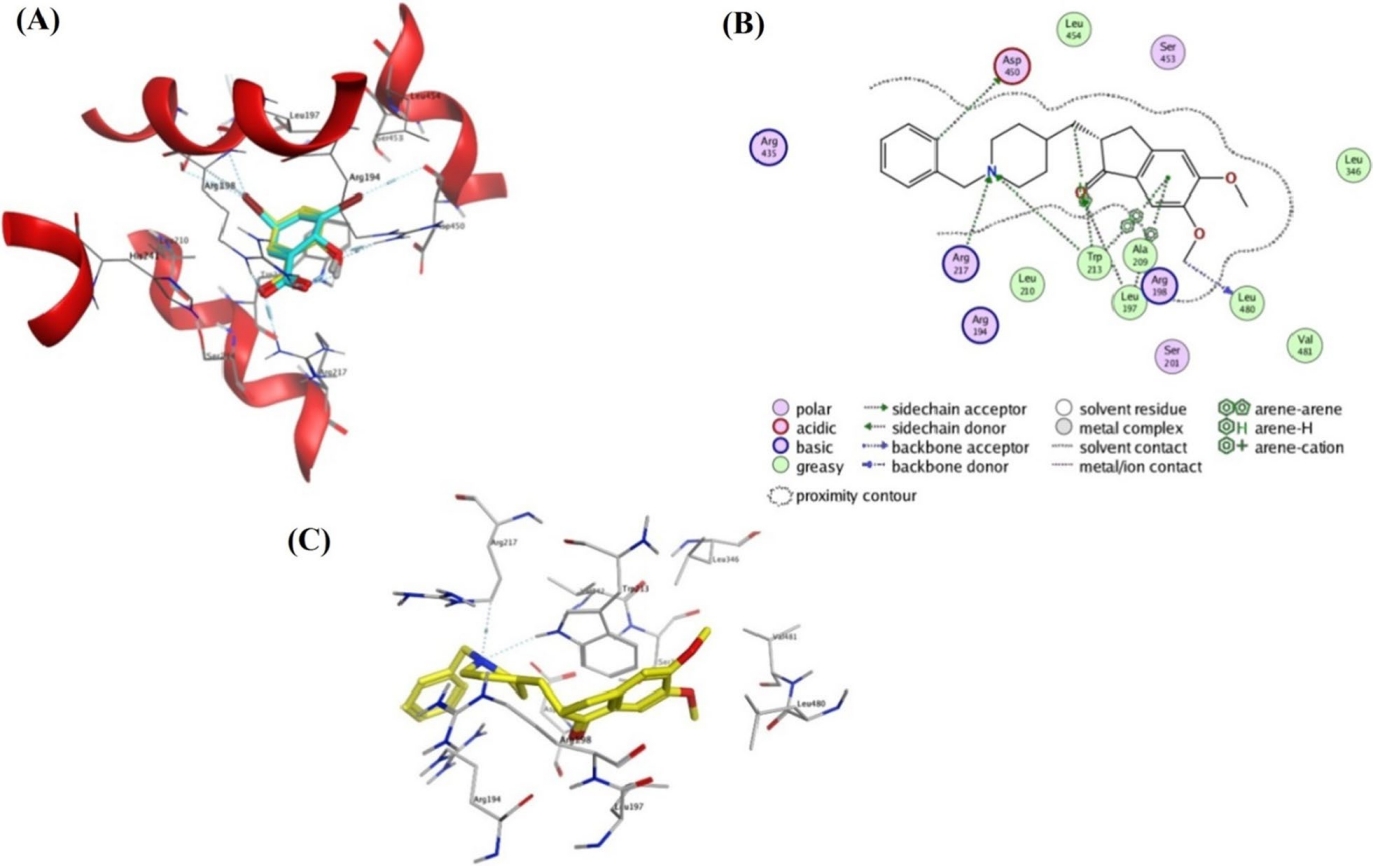
**A** An overlay of the docked pose of DIU (in yellow) that is generated by MOE 2019.0102 with the original one that is deposited in PDB 4JK4 (in cyan). **B** Docking and binding pattern of DNP into BSA active site (PDB 4JK4) in 2D view. **C** Docking and binding pattern of DNP into BSA active site (PDB 4JK4) in 3D view

At this point, the binding of DNP with BSA at the site I of subdomain II was demonstrated by experimental site marker displacement studies. For further confirmation, we opted to utilize molecular docking simulation to provide further theoretical insights into the nature of ligand–protein interactions as well as the possible binding modes. Molecular operating environment software (MOE 2019.0102) was used to run the experiment. The X-ray crystal structure of bovine serum albumin (PDB ID: 4JK4) with its co-crystallized ligand 3,5-diiodosalicylic acid (DIU) was obtained from the protein data bank. (https://www.rcsb.org/structure/4jk4). Four binding sites have been identified for DIU binding with BSA and special interest was drawn toward the elongated cavity of site I of subdomain II that allowed π-stacking interactions with the indole ring of Trp213. The MOE search algorithm and scoring function were used to determine the best binding poses. Validation of the docking protocol was carried out by redocking of the co-crystallized ligand into the above-mentioned site. Retrieval of the native PDB pose occurred with a root mean square deviation (RMSD) of 0.31 and a docking score of − 6.71 kcal/mol (Fig. 12A).

Careful inspection of the binding profile of DNP into BSA site I of Subdomain II revealed a number of hydrophobic interactions and hydrogen bonding. The most important of which was displayed as arene-arene interaction between the key amino acid residue Trp213 and the phenyl portion of the indanone ring. The same residue was also involved in arene-hydrogen interaction with the neighboring CH_2_ group as well as a hydrogen bond with the acceptor piperidine nitrogen. Leu197 formed another hydrophobic contact with the indanone ring. In addition, binding was strengthened owing to the establishment of a number of hydrogen bonds with BSA residues namely Arg217, Asp450, and Leu480 (predicted docking score of − 8.71 kcal/mol) (Fig. 12B and C). Therefore, the results of the docking study were consistent with the experimental data by portraying the potential molecular interactions responsible for the binding of DNP with BSA.

## Conclusion

In the present study, the DNP-BSA binding interaction was evaluated under physiological settings using different spectroscopic and molecular docking methodologies. An intensive study was carried out utilizing different techniques and tools. DNP-BSA interaction was found to be through combined dynamic and static quenching. Additionally, the DNP-BSA interaction's thermodynamic parameters were established. As the result of the negative value of (ΔG^ο^) and positive value of (ΔS^ο^), DNP-BSA binds spontaneously, with hydrophobic interaction predominating in the process. Based on the site marker technique approved using molecular docking methods, DNP-BSA interaction happens at the site I in subdomain IIA. This research contributes to a better knowledge of DNP's pharmacokinetic characteristics. The data revealed from this research may help in the study of the molecular mechanisms driving DNP’s severe adverse effects, hence enhancing its pharmacological and clinical effectiveness.

## Data Availability

Datasets generated and/or analyzed during the current study are available from the corresponding author upon reasonable request.
